# Age-Related Trends in Corneal Refractive Parameters: A Fourier Analysis of Videokeratography in Healthy Eyes

**DOI:** 10.3390/biomedicines13112740

**Published:** 2025-11-10

**Authors:** Shumei Tan, Ziyuan Liu, Yuanting Li, Xiang Li, Yicheng Tong, Xuemin Li

**Affiliations:** 1 Beijing Key Laboratory of Restoration of Damaged Ocular Nerve, Department of Ophthalmology, Peking University Third Hospital, 49 North Garden Road, Haidian District, Beijing 100191, China; 2 Beijing Jishuitan Hospital, Fourth Clinical College of Peking University, 68 Huinan North Road, Changping District, Beijing 102200, China; 3 School of Basic Medical Sciences, Peking University, 38 Xueyuan Road, Haidian District, Beijing 100191, China

**Keywords:** regular astigmatism, irregular astigmatism, Fourier series analysis, cornea

## Abstract

**Purpose**: To investigate the distribution and trend of corneal parameters in different age groups without corneal diseases. **Methods**: A retrospective cross-sectional study. We enrolled 2545 patients (5074 eyes) without corneal diseases (mean age 37.0 ± 26.8, range 4–95) between August 2018 and December 2022. This study aimed to analyze corneal refractive power parameters derived from Fourier transform (minimum spherical refractive power (SphRMin), central regular astigmatism (Asti.Central), peripheral regular astigmatism (Asti.Periph), and irregular astigmatism (Irregul)). The differences in these parameters across age groups were compared, and both linear and nonlinear equations were fitted to model the age-related changes. **Results**: In people without corneal diseases of all ages, the mean values of SphRMin, Asti.Central, Asti.Periph and Irregul were 7.71 ± 0.28, 0.10 ± 0.062, 0.11 ± 0.068, and 0.028 ± 0.019. There are differences in Asti.Central, Asti.Periph, and Irregul among the different age groups (all *p* < 0.001). SphRMin, Asti.Central, Asti.Periph and Irregul were correlated with age (all *p* < 0.001). SphRMin increased before 15 years and decreased after 15 years (*p* < 0.001). Asti.Central increased up to 19 years, decreased between 19 and 60 years, and then increased after 60 years (*p* < 0.001). Asti.Periph increased up to 18 years, decreased from 18 to 48 years, and then increased after 48 years (*p* < 0.001). Irregul increased up to 13 years, decreased from 13 to 30 years, and then increased after 30 years (*p* < 0.001). SphRMin, Asti.Central, Asti.Periph and Irregul were all correlated (all *p* < 0.05). **Conclusions**: We report the mean values of corneal refractive parameters obtained by the Fourier transform in patients without corneal diseases at different ages, its changing trend with age, and important inflection points. This has great clinical implications for the diagnosis of abnormal refractive state corneas and the design of ophthalmic surgery.

## 1. Introduction

Pentacam’s 3D anterior segment diagnosis and analysis system, based on the optical principle of Scheimpflug, has been increasingly applied to the measurement of corneal morphology before ophthalmic surgery, such as corneal refractive surgery and cataract surgery [1,2]. Scheimpflug imaging can provide high-quality topography of the anterior and posterior corneal surfaces, corneal thickness data, and height maps, as well as Zernike analysis and Fourier analysis of corneal refractive power data [1].

Fourier series analysis decomposes circumferential fluctuations in corneal power into components that have direct clinical correlates. The spherical equivalent and regular astigmatism decomposed by Fourier series analysis are analogous to keratometric spherical (equivalent) and astigmatism powers [3], respectively, (shown in Table 1). The other Fourier components have no keratometric equivalent but have distinct clinical value. Decentration refers to a tilt of the cornea relative to the videokeratography axis, while irregular astigmatism manifests as sinusoidal variations in refractive power that cannot be corrected with standard optical interventions, including spectacles or conventional contact lenses [4]. Corneal irregular astigmatism that compromises visual function despite the absence of other vision-limiting pathologies is associated with multiple conditions: keratoconus, pellucid marginal degeneration (PMD), post-LASIK (laser-assisted in situ keratomileusis) ectasia, specific corneal dystrophies/degenerations, and prior penetrating keratoplasty (PK), infection, or trauma [5].

Since the mid-1990s, Fourier analysis has been proven to be an effective method for measuring corneal conditions, especially for irregular astigmatism [6,7,8]. Previous studies have shown that Fourier analysis can detect subtle changes in corneal shape caused by corneal disease or anterior segment surgery, which has been in Fuchs endothelial corneal dystrophy [4], Keratoconus [9,10], dry eye [11], pterygium [12], photorefractive keratectomy [13], cataract surgery [14] and other diseases [15,16] and surgery has been widely used.

Namba H. et al. reported that with advancing age, the cornea undergoes characteristic changes, including increased curvature, elevated prevalence of astigmatism, and a progressive shift in astigmatism axis orientation from with-the-rule (WTR) to against-the-rule (ATR) [17]. However, there is still a lack of studies on the change in corneal parameters from Fourier analysis with age in normal eyes. Understanding the corneal refractive changes with age in normal eyes assessed by Fourier parameters of videokeratography data is of great significance for identifying patients with keratopathy and irregular astigmatism, and can guide the astigmatism correction strategy before surgery. This study aimed to characterize the distribution and age-related trends of Fourier-derived corneal parameters in normal eyes.

## 2. Materials and Methods

### 2.1. Patients

We retrospectively enrolled patients without corneal diseases who underwent Pentacam examinations at Peking University Third Hospital from August 2018 to December 2022. The included eyes were free of ocular pathologies except for mild refractive errors (myopia, hyperopia, or regular astigmatism ≤ 3.0 diopters [D]). The exclusion criteria were as follows: patients with high astigmatism (cylindrical diopter > 3.0D) and irregular astigmatism; patients with corneal disease; patients with trauma history, eye surgery history, family history of glaucoma, and obvious dry eye symptoms; patients who are unable to cooperate or refuse to be examined. All enrolled patients were divided into nine groups according to age: Group 1 (aged: 4–10), Group 2 (aged: 11–20), Group 3 (aged: 21–30), Group4 (aged: 31–40), Group 5 (aged: 41–50), Group 6 (aged: 51–60), Group 7 (aged: 61–70), Group 8 (aged: 71–80), Group 9 (aged: >80). The Peking University Third Hospital Medical Science Research Ethics Committee approved the study protocol (Approval No. M2022809), which adhered to the tenets of the Declaration of Helsinki. The privacy rights of human subjects were observed, and informed consent for participation in the experiments was obtained. Data was analyzed anonymously.

### 2.2. Measurement of Corneal Fourier Parameters

Corneal parameters were measured by Pentacam 70700 (Oculus Optikgeräte GmbH, Wetzlar, Germany). The subjects were seated in a dark room, with their jaws placed on the jaw rest, their forehead pressed against the forehead band, and their eyes wide open to watch the flashing blue light. The same experienced inspector followed the on-screen prompts to aim with a joystick and took the value of the image quality display “OK”. Repeat this three times for each measure to take the best images. Dioptric data of the anterior corneal surface were decomposed using Fourier harmonic analysis. SphRMin, Asti.Central, Asti.Periph and Irregul obtained from the Fourier transform were selected for analysis. SphRMin, the spherical component, which displays the arithmetic mean of all radii of curvature for each ring at 3.0 mm, 5.0 mm, and 7.0 mm (unit: mm). Asti.Central was defined as regular astigmatism at the cornea center 3.5 mm (unit: D (diopter)). Asti.Periph was defined as regular astigmatism ranging from 3.5 mm to 7.0 mm around the cornea (unit: D (diopter)). Irregul was defined as irregularities that cannot be corrected by sphere, cylinder, or prism (unit: D (diopter)) [10].

### 2.3. Statistical Methods

Data were statistically analyzed by SPSS 27.0.1.0 (IBM, Armonk, NY, USA). The Kolmogorov–Smirnov test was used to check the normality of the data. Descriptive data were expressed as mean ± standard deviation. The rate was expressed as a percentage (%). Levene’s test was used to test the homogeneity of variance of the data. One-way ANOVA and the Kruskal–Wallis test were used to compare differences in the distribution of Fourier parameters between groups. Scatter plots and Pearson Correlation Analysis were used to test the correlation between Fourier parameters and age. linear regression and weighted least squares were used to model linear changes in Fourier parameters with age. Restricted cubic splines and correlation heat map were plotted by R software (version 4.0.5). Considering the large sample size in this study, five knots were selected for restricted cubic spline fitting. For multiple comparisons, *p*-values were adjusted using the Benjamini–Hochberg method to control the False Discovery Rate (FDR). Two decimal places were reserved for all data. *p* values (two-sided) below 0.05 were considered to indicate statistical significance.

## 3. Results

A total of 2545 subjects (5074 eyes) were included. Patient age ranged from 4 to 91 years. There were 2529 right eyes (49.8%) and 2545 left eyes (50.2%) (The baseline characteristics of subjects enrolled are shown in Table 2). There was no significant difference in the proportion of eyes in different age groups ($\chi^{2}$= 0.374, *p* > 0.05, Padj > 0.05 (Padj: the *p*-value adjusted by FDR (False Discovery Rate) using the Benjamini–Hochberg method)).

### 3.1. Mean and Range of Corneal Refractive Parameters in Normal Eyes at Different Ages

**Figure 1:**
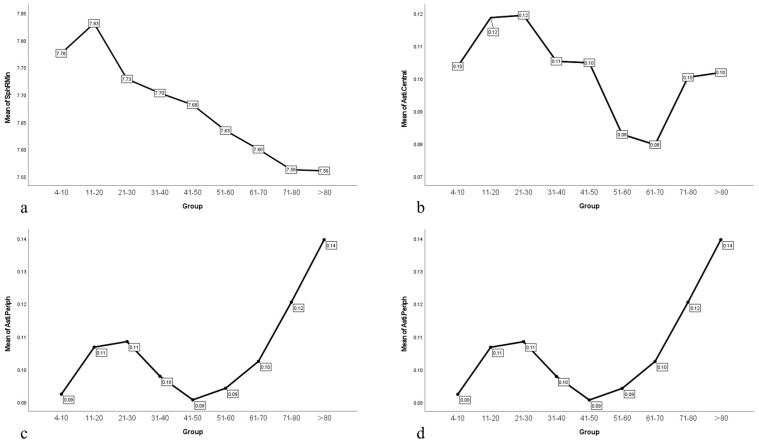
(**a**) Broken line chart of SphRMin with age in normal eyes; (**b**) Broken line chart of Asti.Central with age in normal eyes; (**c**) Broken line chart of Asti.Periph with age in normal eyes; (**d**) Broken line chart of Irregul with age in normal eyes. SphRMin: the spherical component, which displays the arithmetic mean of all radii of curvature for each ring at 3.0 mm, 5.0 mm, and 7.0 mm; Asti.Central was defined as regular astigmatism at the cornea center 3.5 mm. Asti.Periph was defined as regular astigmatism ranging from 3.5 mm to 7.0 mm around the cornea. Irregul was defined as irregularities that cannot be corrected by a sphere, cylinder, or prism.

In normal eyes of all ages, the mean values of SphRMin, Asti.Central, Asti.Periph and Irregul were 7.71 ± 0.28, 0.10 ± 0.062, 0.11 ± 0.068, and 0.028 ± 0.019. The Fourier parameter values of normal eyes in different age groups are shown in Table 3 and Figure 1.

One-way ANOVA for SphRMin showed that there were significant differences in SphRMin between different age groups (F = 88.026; *p* < 0.001; Padj < 0.05). For Asti.Central, Asti.Periph, and Irregul, the homogeneity of variance test showed that the variances were heterogeneous (all *p* < 0.001; all Padj < 0.005). The Welch’s test (an asymptotic F-test) was used to compare differences between groups, and it indicated that for Asti.Central, Asti.Periph, and Irregul, there were significant differences between different age groups (all *p* < 0.001; all Padj < 0.005).

### 3.2. Corneal Refractive Changes with Age in Normal Eyes Assessed by Fourier Analysis

**Figure 2:**
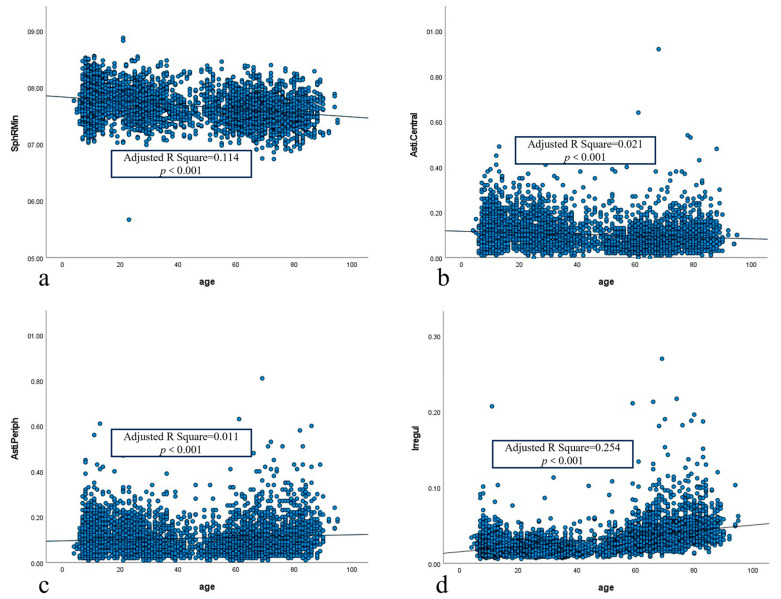
(**a**) Scatterplot of SphRMin with age in normal eyes; (**b**) Scatterplot of Asti.Central with age in normal eyes; (**c**) Scatterplot of Asti.Periph with age in normal eyes; (**d**) Scatterplot of Irregul with age in normal eyes. SphRMin: the spherical component, which displays the arithmetic mean of all radii of curvature for each ring at 3.0 mm, 5.0 mm, and 7.0 mm; Asti.Central was defined as regular astigmatism at the cornea center 3.5 mm. Asti.Periph was defined as regular astigmatism ranging from 3.5 mm to 7.0 mm around the cornea. Irregul was defined as irregularities that cannot be corrected by a sphere, cylinder, or prism.

The scatter plots of SphRMin, Asti.Central, Asti.Periph and Irregul with age are shown in Figure 2. After Pearson Correlation Analysis, it was found that SphRMin, Asti.Central, Asti.Periph and Irregul were correlated with age (all *p* < 0.001; all Padj < 0.005). The results of ANOVA showed statistical significance for the linear fit model of SphRMin, Asti.Central, Asti.Periph and Irregul (all *p* < 0.001; all Padj < 0.005). However, the Adjusted R Square of SphRMin, Asti.Central, Asti.Periph and Irregul were 0.114, 0.021, 0.011, and 0.254, respectively. This indicates that the above linear-fitting models cannot well account for the change in Fourier parameters with time. Therefore, restricted cubic splines were used to further analyze the relationship between the Fourier parameters and age.

**Figure 3:**
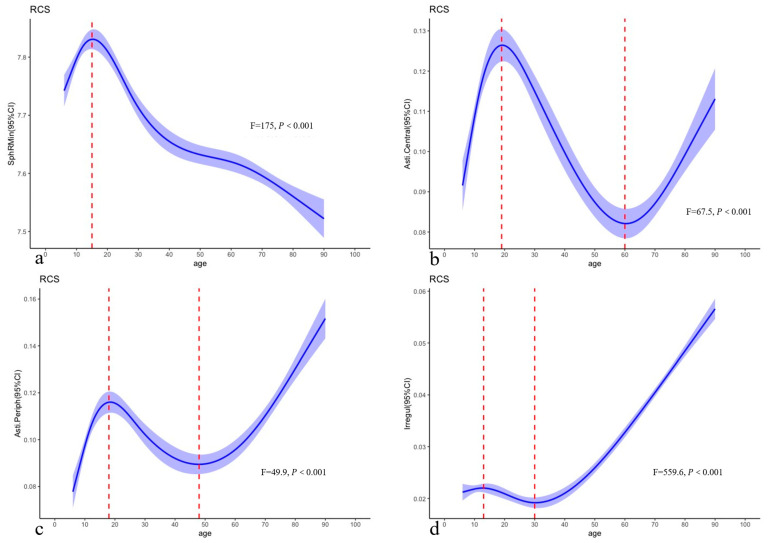
(**a**) 5-knot restricted cubic splines of SphRMin with age in normal eyes; (**b**) 5-knot restricted cubic splines of Asti.Central with age in normal eyes; (**c**) 5-knot restricted cubic splines of Asti.Periph with age in normal eyes; (**d**) 5-knot restricted cubic splines of Irregul with age in normal eyes. SphRMin: the spherical component, which displays the arithmetic mean of all radii of curvature for each ring at 3.0 mm, 5.0 mm, and 7.0 mm; Asti.Central was defined as regular astigmatism at the cornea center 3.5 mm. Asti.Periph was defined as regular astigmatism ranging from 3.5 mm to 7.0 mm around the cornea. Irregul was defined as irregularities that cannot be corrected by a sphere, cylinder, or prism.

Restricted cubic splines of SphRMin showed that SphRMin increases before 15 years and decreases after 15 years. At 15 years, the corresponding SphRMin value was 7.84 (95% CI: 7.77–7.89) (F = 175.0; *p* for nonlinearity < 0.001; Padj for nonlinearity < 0.005) (Figure 3). Restricted cubic splines of Asti.Central showed that it increases up to 19 years, decreases between 19 and 60 years, and then increases again after 60 years (F = 67.5; *p* for nonlinearity < 0.001; Padj for nonlinearity < 0.005). The corresponding Asti.Central values are 0.13 (95% CI: 0.12–0.13) at 19 years and 0.082 (95% CI: 0.078–0.086) at 60 years. Restricted cubic splines of Asti.Periph showed that Asti.Periph increased up to 18 years, decreased from 18 to 48 years, and then increased again after 48 years (F = 49.9; *p* for nonlinearity < 0.001; Padj for nonlinearity < 0.005). At 18 years, the corresponding Asti.Periph value was 0.12 (95% CI: 0.11–0.12); at 48 years, it was 0.089 (95% CI: 0.083–0.095). Restricted cubic splines of Irregul showed that Irregul increased up to 13 years, decreased from 13 to 30 years, and then increased again after 30 years (F = 559.6; *p* for nonlinearity < 0.001; Padj for nonlinearity < 0.005). At 13 years, the corresponding Irregul value was 0.022 (95% confidence interval [95%CI]: 0.021–0.023); at 30 years, it was 0.019 (95%CI: 0.018–0.020).

### 3.3. Correlation Between Corneal Refractive Parameters Obtained by Fourier Analysis

**Figure 4:**
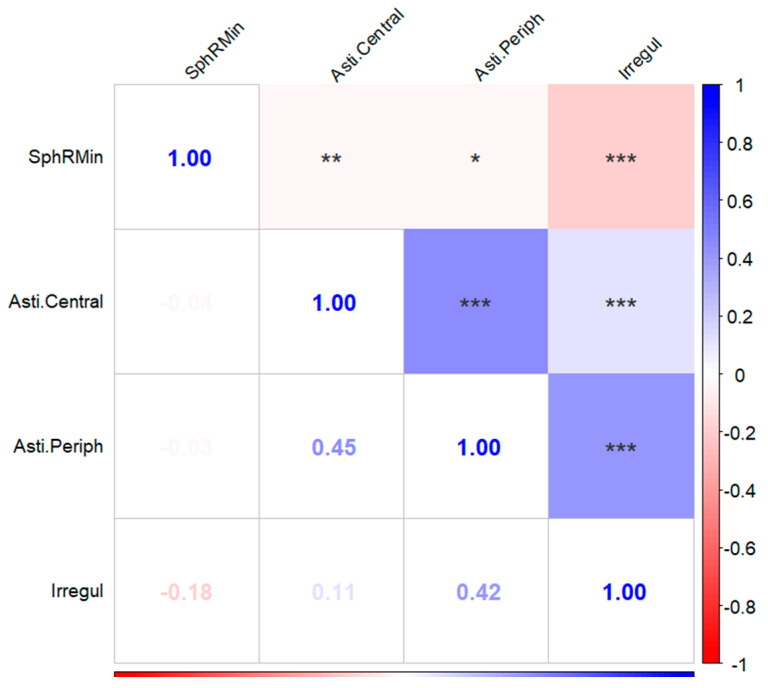
Associations between corneal refractive parameters based on Fourier analysis. SphRMin: the spherical component, which displays the arithmetic mean of all radii of curvature for each ring at 3.0 mm, 5.0 mm, and 7.0 mm; Asti.Central was defined as regular astigmatism at the cornea center 3.5 mm. Asti.Periph was defined as regular astigmatism ranging from 3.5 mm to 7.0 mm around the cornea. Irregularity was defined as irregularities that cannot be corrected by a sphere, cylinder, or prism. Coefficient of correlation of SphRMin, Asti.Central, Asti.Periph and Irregul are presented by a coloring scheme from red (negative correlation) to blue (positive correlation), while white represents an absence of correlation. *: *p* < 0.05; **: *p* < 0.01; ***: *p* < 0.001.

Pearson Correlation Analysis showed that SphRMin, Asti.Central, Asti.Periph and Irregul were all correlated (all *p* < 0.05; all Padj < 0.05). SphRMin and Asti.Central, SphRMin, and Asti.Periph, SphRMin, and Irregul were negative correlations (Pearson correlation coefficients were −0.040, −0.034, and −0.18). Asti.Central and Asti.Periph, Asti.Central and Irregul, Asti.Periph and Irregul were positive correlations (Pearson correlation coefficients were 0.45, 0.11, and 0.42) (shown in Figure 4).

## 4. Discussion

Corneal morphology and refractive status change with age. Anterior and posterior corneal curvature (K1 and K2) measured by pentacam increased with age in Asian healthy eye subjects, but corneal astigmatism (ΔK) on the anterior and posterior surfaces of the cornea and central, minimum, and global corneal thickness decreased with age [18]. E. Karmiris et al. [19] found that corneal densitometry increased with age using Scheimpflug camera evaluation. A comprehensive understanding of the distribution of corneal refractive parameters after Fourier transform in different age groups is of great significance for the diagnosis and identification of corneal abnormal diseases and the design of various refractive surgeries, and is conducive to the evaluation of postoperative visual quality. Our study is the first to analyze the corneal refractive parameters based on Fourier analysis in a large sample of 4–91-year-olds with normal eyes and to explore their changing trends with age.

TANABE T et al. [20] showed that the Fourier parameters of the normal eyes based on videokeratography (TMS-2) ranged from 0 to 1.04 D for regular astigmatism, from 0.02 to 0.68 D for asymmetry, and from 0.05 to 0.17 D for higher-order irregularity. In the study by ASGARI S et al., the average horizontal and vertical radius of corneal curvature measured by Pentacam in normal people aged 40 to 64 years was 7.77 ± 0.52 mm and 7.68 ± 0.47 mm, respectively [21]. In the study by H. Sideroudi et al., the values of SphRMin, Asti.Central, Asti.Periph and Irregul in the normal eye population were 7.792 ± 0.2819, 0.116 ± 0.05273, 0.0818 ± 0.05271, and 0.02 ± 0.007073. In our study, the mean values of SphRMin, Asti.Central, Asti.Periph, and Irregul in normal eyes were 7.71 ± 0.28, 0.10 ± 0.062, 0.11 ± 0.068, and 0.028 ± 0.019.

We found that the spherical component, regular astigmatism at the center, regular astigmatism at the periphery, and irregular astigmatism are all associated with age. Mehdi KhabazKhoob et al. [22] reported age, spherical equivalent, and corneal diameter were significantly correlated with mean keratometry reading in a normal population at Tehran. Additionally, mean corneal irregularity in the central 3 mm zone was 1.25D, which was significantly correlated with age.

Age has been proven to be a crucial factor influencing corneal biomechanics [1]. Guihua Liu et al. investigated the distribution of the corneal tissue material stiffness parameter SSI across different age groups and its associated factors in a healthy Chinese population. Their study revealed that SSI increases with age after 35 years old [2]. Daxer and Malik, through corneal X-ray examinations, observed that non-enzymatic crosslinking, collagen glycation, fibril diameter, and the number of collagen molecules increase with age in individuals over 40 years old [3]. This may explain the findings in our study that Asti.Central and Asti.Periph increases with age after 60 years old, while Irregul increases with age after 30 years old. From adolescence to early adulthood (13–30 years), tear film stability peaks [23], supported by a well-functioning lipid layer and frequent, complete blinks that ensure uniform corneal wetting—this minimizes surface irregularities, explaining the observed decrease in Irregul during this phase. After 30 years of age, progressive decline in meibomian gland secretion and tear film stability leads to focal dry spots and epithelial desiccation [24], which increases corneal surface roughness and directly drives the gradual elevation of Irregul observed in our study; for elderly individuals (≥60 years), the cumulative effects of tear film instability—exacerbated by reduced epithelial repair capacity—further amplify irregular astigmatism and contribute to the rebound of regular astigmatism. The age-related corneal refractive norms (SphRMin, Asti.Central, Asti.Periph, Irregul) from our study guide personalized ophthalmic surgery. For cataract surgery, ≥60-year-olds with Asti.Central/Asti.Periph exceeding age-specific may need repeat keratometry to avoid IOL calculation errors [25].

Irregular astigmatism cannot be corrected with glasses [26]. RGP (Rigid Gas Permeable Contact Lens) is the gold standard for the non-surgical treatment of patients with corneal ectasia and irregular astigmatism. RGP has some problems, such as poor correction effect in patients with severe corneal irregularity or intolerance in patients with irregular astigmatism [27]. Therefore, the early identification of irregular astigmatism is particularly important. A previous study showed that corneal irregularity increased with age in both sexes [28]. However, our study found that irregular astigmatism increased first, then decreased, and then increased with age in a nonlinear manner.

Yuta Ueno et al. [29] investigated the relation between corneal regular and irregular astigmatism in normal human eyes. They found that the pattern of regular corneal astigmatism had a significant effect on the amount of irregular corneal astigmatism (*p* < 0.001). In our study, we found that the four Fourier parameters included in the study were SphRMin, Asti., Central, Asti.Periph, and Irregul were all correlated with each other, but Asti.Central and Asti.Periph, Asti.Periph and Irregul were most correlated (Pearson correlation coefficients were 0.45 and 0.42, respectively).

Previous studies had studied the association of CDVA (corrected distance visual acuity) and Fourier components of the anterior and posterior corneal surfaces in patients with Fuchs Endothelial Corneal Dystrophy [4]. They found that higher spherical power of the anterior and posterior corneal surfaces was associated with poorer CDVA. But there were no significant associations between CDVA and regular astigmatism, higher-order irregularity at 3 and 6 mm. SHIKA T et al. showed that the Fourier irregular astigmatism component is significantly correlated with spectacle-corrected visual acuity, while other corneal topography-related indices, such as surface regularity index and surface asymmetry index, are not [30]. However, in our study, the Fourier irregular astigmatism component was correlated with age.

This study has several limitations. Firstly, it adopts a retrospective cross-sectional design, which only captures static snapshots of corneal Fourier parameters across different age groups rather than tracking dynamic changes within individual subjects over time. This prevents distinguishing whether the observed age-related trends reflect true intra-individual aging effects or inherent differences between age cohorts. Secondly, the hospital-based sample introduces potential referral bias, as participants were seeking care for refractive errors, cataracts, or other ocular concerns, potentially overrepresenting individuals with subclinical tear film dysfunction or mild corneal irregularities compared to the general healthy population. Thirdly, sample sizes were relatively small for extreme age groups (under 10 years and over 80 years), limiting the representativeness of normative values for these segments. Additionally, key clinical correlates were not included, such as corrected distance visual acuity (CDVA), tear film metrics (e.g., break-up time), corneal biomechanical parameters, gender, and refractive error types, which hindered exploration of their regulatory roles in parameter trends and confounding effects. These limitations underscore the need for future multi-center prospective cohort studies with long-term follow-up, community-based sampling to reduce bias, and integration of comprehensive clinical and microstructural assessments (e.g., OCT, Corvis ST) to validate findings and clarify underlying mechanisms.

## Figures and Tables

**Table 1:** The clinical descriptions of the components generated by Fourier series analysis of corneal topography data.

Spherical equivalent	Zero frequency component	*n* = 0
Decentration	One-cycle component	*n* = 1
Regular astigmatism	Two-cycle component	*n* = 2
Irregular astigmatism	Higher-order components	*n* = 3

**Table 2:** Baseline Characteristics of subjects enrolled.

Group	1	2	3	4	5	6	7	8	9			
Age range	1–10	11–20	21–30	31–40	41–50	51–60	61–70	71–80	81–95	$\chi^{2}$	*p* value	Padj value *
n	488	549	337	169	73	176	351	243	168			
Eyes	971	1095	674	335	145	348	699	479	328			
Right eyes/all eyes	50.3%	49.9%	50.0%	49.6%	50.3%	49.4%	50.2%	49.3%	48.8%	0.374	1.00	1.00

* The *p*-value adjusted by FDR (False Discovery Rate) using the Benjamini–Hochberg method.

**Table 3:** Mean and range of corneal refractive parameters based on Fourier analysis of normal eyes at different ages.

Group	*n*	SphRMin (mm)		Asti.Central (D)		Asti.Periph (D)		Irregul (D)	
		Mean ± SD	Range	Mean ± SD	Range	Mean ± SD	Range	Mean ± SD	Range
1	971	7.78 ± 0.26	7.03–8.53	0.10 ± 0.053	0.01–0.39	0.092 ± 0.057	0.01–0.45	0.022 ± 0.0099	0.0060–0.10
2	1095	7.83 ± 0.25	7.03–8.56	0.19 ± 0.061	0.01–0.49	0.11 ± 0.063	0.01–0.61	0.022 ± 0.011	0.0060–0.21
3	674	7.73 ± 0.28	5.67–8.88	0.12 ± 0.064	0.00–0.41	0.11 ± 0.063	0.01–0.47	0.020 ± 0.0083	0.0060–0.086
4	335	7.70 ± 0.26	7.07–8.48	0.11 ± 0.05	0.01–0.36	0.098 ± 0.063	0.01–0.39	0.020 ± 0.0096	0.0070–0.11
5	145	7.68 ± 0.28	7.11–8.39	0.10 ± 0.066	0.01–0.38	0.09 ± 0.059	0.01–0.34	0.022 ± 0.013	0.0080–0.10
6	348	7.63 ± 0.27	6.98–8.46	0.083 ± 0.058	0.01–0.40	0.09 ± 0.064	0.01–0.51	0.029 ± 0.017	0.0080–0.21
7	699	7.60 ± 0.26	6.75–8.39	0.080 ± 0.061	0.00–0.92	0.10 ± 0.073	0.01–0.81	0.038 ± 0.022	0.0090–0.27
8	479	7.56 ± 0.28	6.74–8.40	0.10 ± 0.068	0.01–0.54	0.12 ± 0.082	0.01–0.53	0.046 ± 0.025	0.013–0.22
9	328	7.56 ± 0.26	6.92–8.29	0.10 ± 0.065	0.01–0.48	0.14 ± 0.087	0.01–0.60	0.050 ± 0.023	0.015–0.19
Total	5074	7.71 ± 0.28	5.67–8.88	0.10 ± 0.062	0.00–0.92	0.11 ± 0.068	0.01–0.81	0.028 ± 0.019	0.0060–0.27

SphRMin: the spherical component, which displays the arithmetic mean of all radii of curvature for each ring at 3.0 mm, 5.0 mm, and 7.0 mm; Asti.Central was defined as regular astigmatism at the cornea center 3.5 mm. Asti.Periph was defined as regular astigmatism ranging from 3.5 mm to 7.0 mm around the cornea. Irregul was defined as irregularities that cannot be corrected by a sphere, cylinder, or prism. Group 1 (aged:1–10), Group 2 (aged: 11–20), Group 3 (aged: 21–30), Group 4 (aged: 31–40), Group 5 (aged: 41–50), Group 6 (aged: 51–60), Group 7 (aged: 61–70), Group 8 (aged: 71–80), Group 9 (aged: >80). (c) SD: standard deviation.

## Data Availability

The data supporting the reported results can be found in Mendeley Data, V2, doi: 10.17632/yr8hbwmsvy.2.
